# Aetiology, Risk Factors, Diagnosis and Control of Foot-Related Lameness in Dairy Sheep

**DOI:** 10.3390/ani9080509

**Published:** 2019-07-31

**Authors:** Athanasios I. Gelasakis, Aphrodite I. Kalogianni, Ioannis Bossis

**Affiliations:** Laboratory of Anatomy and Physiology of Farm Animals, Department of Animal Science and Aquaculture, Agricultural University of Athens (AUA), Iera Odos 75 str., 11855 Athens, Greece

**Keywords:** contagious ovine digital dermatitis, control, dairy sheep, *Dichelobacter nodosus*, foot-related lameness, footrot, milk production, risk factors, scald, white line disease

## Abstract

**Simple Summary:**

Despite improvements in modern livestock management, intensification of farming systems has led to the emergence of infectious and husbandry-related health and welfare challenges. Among them, foot pathology-associated lameness is one of the most significant, as it has been strongly associated with adverse consequences in the well-being of animals, and the productivity and the overall profitability of the farms. The objective of this article is to review the current status of knowledge regarding foot-related lameness in sheep, emphasizing on the etiology, risk factors, diagnostic capabilities and control strategies of foot problems in dairy sheep.

**Abstract:**

During the last twenty years, considerable research efforts have recognized the consequences of foot-related lameness primarily in cattle, and meat and wool sheep. Despite the lack of extensive epidemiological studies, field observations and isolated research reports in dairy sheep have suggested that the problem might be more severe in semi-intensive and intensive farming systems. Footrot, contagious ovine digital dermatitis, ovine interdigital dermatitis, white line disease, and pedal joint abscess are the most common causes of foot-related lameness. *Dichelobacter nodosus*, *Fusobacterium necrophorum*, *Treponema* spp., and *Actinomyces pyogenes* are the most significant foot-related lameness-associated pathogens. Despite a documented hereditary predisposition, environmental factors are the most important in determining the occurrence of foot-related lameness. Moist and warm environment, increased parity and milk yield, inappropriate housing conditions and infrastructures, inadequate hygiene status, imbalanced nutrition, and insufficient foot care are the most critical risk factors. Furthermore, a foot-lameness control plan should include targeted implementation of claw trimming and footbathing, evidence-based planning of hygiene measures in preventive veterinary practices (i.e., antibiotic administration, vaccinations against footrot), selective breeding to footrot resistance, and, most importantly, the continuous training of farming personnel. Controlling foot-lameness in dairy sheep is critical in determining the well-being of animals, and strongly affects the farm’s profitability and sustainability.

## 1. Introduction

Sheep farming is one of the earliest agricultural activities worldwide [1,2]. It has evolved through centuries, from the extensive to the semi-extensive system, mainly in Europe, Asia, and Africa following migratory population patterns and socioeconomic evolution of rural areas. In recent years, the increasing global demand for sheep dairy products has resulted in the rapid intensification of farming systems, particularly in the European countries with the most developed dairy sheep sector (e.g., Greece, Italy, France, Spain, and Romania). As a result, semi-intensive and intensive systems have been established and continuously evolved, reshaping the sector in these regions. Consequently, the required inputs and the expected outputs have increased, genetic selection of animals has been intensified, and infrastructures, equipment, and modern technologies have been integrated as part of more sophisticated management systems. Despite improvements in animal management, specific husbandry-oriented health and welfare challenges have emerged. Among them, foot pathology-associated lameness is considered one of the most significant. It has been strongly associated with adverse consequences in the well-being of animals, and the productivity and the overall profitability of dairy sheep farms [3].

Considerable research efforts have focused on the investigation of foot-related lameness and its epizootiology, pathogenesis, diagnostic capabilities, and the impact on production, health and welfare status, as well as on the development of effective treatments and application of sustainable preventive measures. The majority of relevant research efforts have focused primarily on cattle [4,5,6,7,8,9,10,11], meat and wool sheep [12,13,14,15], and dairy goats [16,17], highlighting the impact of foot-related lameness on several production traits in these species [12,13,14,15]. Although relevant studies in dairy sheep are limited, recent evidence has suggested a detrimental effect of foot-related lameness on both their well-being and productive efficiency [18,19,20]. Foot-related lameness in sheep is observed in all production systems. However, the risk factors, epizootiology, and effective control strategies may differ depending on the production system. Therefore, universal extrapolation of knowledge and information from meat and wool sheep in dairy sheep, and vice versa, may not always be appropriate.

In the present article, the most recent literature on foot-related lameness in dairy sheep is reviewed. Emphasis is placed on the etiology, risk factors, diagnosis, and control strategies of foot pathologies in dairy sheep. The description of systemic diseases that may manifest lameness among other clinical symptoms is beyond the scope of this review.

## 2. Etiology of Foot-Related Lameness

Lameness is the deviation from normal gait, accompanied or not by pain and distress. It is a sign rather than a disease itself, usually caused by injuries, lesions, defects, and diseases affecting the limbs, the joints, or other parts of the body [3,21,22,23]. Several etiological factors are associated with lameness in sheep. For example, it may occur as a result of nervous and musculoskeletal disorders [12], or as a clinical manifestation of notifiable systemic viral diseases causing inflammation in the region of the coronary band, such as contagious ecthyma [24], foot-and-mouth disease [25,26], or bluetongue [27,28]. However, in the majority of cases, lameness is associated with bacterial infections, lesions, and diseases located at the foot [29,30] and characterized as foot-related lameness. The main causes of foot-related lameness in sheep are common, regardless of their productive orientation (meat-, wool-, or milk-production), as described in detail below.

### 2.1. Footrot

Footrot is a chronic, infectious, and potentially very contagious disease of the foot. It initially infects the skin of the interdigital space and subsequently, the rest of the hoof, with debilitating effects on its integrity [31]. It is caused by the synergistic action of two anaerobic Gram (-) bacteria, namely *Fusobacterium necrophorum* and *Dichelobacter nodosus* [12,32]. *D. nodosus* is the primary causative agent and a necessary component of the disease [33], whereas, the presence of *F. necrophorum* is considered an auxiliary element that influences the predisposition and the establishment of the disease [32]. Depending on the virulence of *D. nodosus* strains, clinical manifestation of footrot may vary from a mild disease (benign footrot) to a more severe one (virulent footrot). Except for *F. necrophorum* and *D. nodosus*, other bacteria may also be implicated in the pathogenesis of the disease, such as *Bacteroides fragilis*, *Prevotella* spp. and *Treponema* spp.; these organisms have been isolated from footrot cases, but their role remains controversial [34]. Footrot causes lameness of classified severity depending on the location and the extent of lesions, and according to the progression of the disease. At early stages, it is characterized by limited inflammation of the tissues at the interdigital space. In these cases, lameness is mild and not always apparent, but the transmission of causative bacteria is favored contributing in footrot dispersion within the flock [31]. Subsequent stages are characterized by the underrunning of the claw horn, which can progressively become totally separated from the underlying tissues at advanced stages of the disease. In that case, the interdigital skin and the underlying hoof matrix are necrotized, pungent, and putrid [12,31]. Epizootiological characteristics of footrot are dependent on microbial (virulence of strains), environmental (moisture and temperature), and host genetic factors (resistance or susceptibility to *D. nodosus*) [12,31,35].

### 2.2. Ovine Interdigital Dermatitis (OID)-Scald

Ovine interdigital dermatitis (OID) is caused by *F. necrophorum*, an opportunistic pathogen typically found in the natural microflora of the digestive tract, which under certain conditions can cause inflammation and relatively mild and superficial lesions in the interdigital skin disrupting its integrity [12,31]. The clinical signs of OID include exudative inflammation and the loss of hair on the interdigital skin, without the underrunning of claw horn, which differentiates it from the benign footrot and the early stages of virulent footrot [12].

Scald is an inadequately defined term, describing infection and inflammation limited to the interdigital space [36]. By definition, scald is not a distinct entity and may refer to OID, benign footrot, or the early stages of virulent footrot [37].

### 2.3. Contagious Ovine Digital Dermatitis (CODD)

Contagious ovine digital dermatitis (CODD) was firstly reported in the UK in 1997. Until then, CODD cases were likely misdiagnosed as severe footrot. More recently, a prevalence rate of ca. 50% has been reported among the sheep farms in the country [38]. The spirochaete *Treponema* spp. and the bacteria *D. nodosus* and *F. necrophorum* are considered to be the principal agents involved in the pathogenesis of the disease; however, the primary causative agent has yet to be elucidated [38,39,40,41]. In any case, similarities between treponemes isolated from CODD and bovine digital dermatitis (BDD) cases [41,42,43] may indicate a potential primary causative role of treponemes in CODD [41]. The clinical manifestation of CODD is different compared to footrot. In the majority of CODD cases, the initial skin lesions are located in the coronary band rather than in the interdigital space as in the cases of footrot. At later stages of CODD though, the inflammation expands in the underlying tissues of the hoof, ending up with a separation of the claw horn which is followed by severe lameness [12,38,40].

### 2.4. White Line Disease (WLD)

The etiology of white line disease (WLD) in sheep is unknown, but seems unlikely to be infectious [12,44]. Factors such as the type and condition of ground and bedding, nutritional deficiencies, and genetic factors have been proposed as potential etiological factors [44]. WLD in sheep is presented as a discolored region of the claw horn at the junction between the hoof wall and the hoof sole, and/or separation of the hoof wall from the laminar corium. In severe cases, the latter lesion progresses into a cavity, also known as ‘shelly hoof’, which is filled with organic matter; this creates the ideal conditions for the proliferation of anaerobic bacteria and the development of inflammation and abscesses, with exudation of pus in chronic cases (white line abscesses) [12,44]. WLD manifestation can vary from limited lesions in the white line without lameness, to extended detachment of claw horn from the hoof wall and sole with pus exudation from the coronary band (white line abscess or toe abscess) [44].

### 2.5. Pedal Joint Abscess

A pedal joint abscess usually occurs in one digit and is the result of infection by *F. necrophorum* and *Actinomyces pyogenes* [12,36]. The joint infection can originate from lesions in the interdigital skin, from white line abscesses, and from bacteraemia [12]. Interdigital dermatitis favors the invasion of bacteria and the infection of the adjacent interphalangeal joint [45]. The hoof is swollen, hot, and extremely painful, with pus exudating from the interdigital space and/or the coronary band after pressure. Severe lameness is the characteristic symptom both in acute and in the chronic phase of the disorder [36].

### 2.6. Laminitis

Laminitis is an aseptic inflammation of the laminar corium in the hooves of sheep, resulting from a disruption in the blood circulation of the foot after ruminal acidosis, toxaemia, or severe infection. In dairy cattle, there is evidence of a correlation between rumen acidosis and laminitis, caused mainly by increased consumption of concentrates and decreased roughages [46]. Laminitis due to ruminal acidosis is uncommon in meat and wool sheep. Its occurrence in dairy sheep has not been investigated thoroughly; however, it is likely to be higher due to the increased consumption of concentrates and the commonly disrupted roughages to concentrates ratio in high producing flocks [23,47].

### 2.7. Granulomas

Granulomas are uncommon in dairy sheep and the available literature is scarce. Granulomatous tissue develops from the lamina corium after its injury (excessive foot-trimming) and it may or not be covered by claw horn. Granulomas may lead to chronic lameness and predispose to infections by *D. nodosus* [12].

### 2.8. Less Common Causes of Foot-Related Lameness

Accumulatiοn of grass, mud, and manure in feet may cause the impaction of interdigital space (soil balling) or the occlusion of the interdigital gland, leading to foot-related lameness [12,23,48]. Other less common causes of lameness include injuries and foreign bodies, ergotism (due to the toxins ergotoxine, ergotamine, ergometrine, ergocornine, ergocristine, and ergosine produced by the ergot fungus Claviceps purpurea) [49], fescue toxicosis with gangrenous necrosis and sloughing of the hoof (caused by ergovaline and lolitrem B toxins found in endophyte-infected tall fescue and perennial ryegrass, respectively) [50,51], dermatophilosis or strawberry footrot caused by *Dermatophilus congolensis* [52], and post-dipping lameness in adult sheep caused by *Erysipelothrix rhusiopathiae* [53].

## 3. Risk Factors of Foot-Related Lameness

Although the etiological factors of foot-related lameness are common among the meat, wool, and dairy sheep breeds, their epizootiology (predisposing factors, prevalence, incidence rate etc.) and control may differ depending on the productive direction of the breed. Differences among these breeds in production systems, feeding regimes, available infrastructures, husbandry practices, hygiene measures, and flock health management schemes may offer an explanation. Irrespective of the etiology of foot-related lameness, the risk factors associated with its occurrence can be either genetic or environmental and interact between them [23].

### 3.1. Genetic Factors

The exploitation of the host’s genetic resistance to footrot has been demonstrated in meat and wool breeds, mainly in the United Kingdom (UK), Australia, and New Zealand [54,55]. In these studies, moderate heritability was evident, especially in flocks affected by footrot. However, the heritability estimates were skewed in flocks with low prevalence due to continuous removal of affected sheep for many years. Thereby, it was concluded that exposure to the disease is critical for the quantification of the likelihood to successfully breed for resistance to footrot [56]. To overcome this limitation, molecular genetic screening is an alternative option for the efficient determination of resistance or susceptibility to the disease. In that case, the exposure to footrot is not a prerequisite to classifying animals as susceptible or resistant, while, the time, labour, and expertise required for the identification and the detailed assessment of footrot lesions are minimized. For molecular screening, the use of extensively polymorphic genetic markers has been suggested [57]. These markers have been identified on the chromosome 20 at the DQA2 and DQA2-like loci in the ovine Major Histocompatibility Complex (MHC-ovar) genes, which have been found to be associated with various degree of susceptibility/resistance to footrot [19,23,58] via modulation of the cellular immune response against *D. nodosus* [57,59,60]. It is well known in most vertebrates that the loci of MHC genes are the most polymorphic of any chromosomal region. The MHC complex of proteins regulates immune responses, and it is one of the most important chromosomal regions affecting resistance or susceptibility to several diseases, especially infectious diseases. Among the DQA2 haplotypes, haplotypes G (0101–1401) and J2 (0702–1401) were associated with resistance to footrot, whereas alleles E (1101) and L (0501) were associated with susceptibility to it [61]. The susceptibility to footrot of animals carrying allele E (1101) has also been confirmed in dairy sheep [62].

Beyond the MHC-DQA2 loci, a recent genome-wide association study [(using the ovine 50 K SNP (single nucleotide polymorphisms) array] investigating susceptibility to footrot in Texel sheep identified seven potential SNPs linked to footrot resistance. However, no significant QTL(quantitative trait loci) could be revealed due to chromosomal imbalance of SNPs in the 50 K array [63].

A genetic basis in the occurrence of WLD due to the hoof keratin structure in Scottish Blackface and Texel sheep has been suggested [64]. In addition, the phenotype of black-colored hooves, found in some breeds (e.g., Chios dairy breed), has been linked to foot-related lameness resistance. In contrast, waxy-colored hooves have been linked to foot-related lameness susceptibility.

### 3.2. Environmental Factors

#### 3.2.1. Season

In dairy sheep, a seasonal variation in manifestation of lameness is evident. This variation is associated not only with the climate conditions, but also with the seasonal pattern of their production cycle. For example, lambing and the first stage of lactation coincide with the wet season (autumn), when relatively warm weather, increased moisture, and extended housing periods predispose to the proliferation and transmission of pathogens. Accumulated infectious pressure, coinciding with a potential failure to meet the increased metabolic demands of the aforementioned stages, may predispose to diseases and lesions associated with foot-related lameness.

#### 3.2.2. Physiological Factors

Among physiological factors, parity number and stage of lactation have been identified as risk factors for foot-related lameness in dairy sheep [19]. Multiparous dairy ewes are at a higher risk of developing foot-related lameness comparing to primiparous (from ca. 6.5 to 19.0 times), whereas, the incidence of foot-related lameness is likely higher during the first three months of lactation [19]. Increased milk yield in adult ewes during the early stages of lactation increases their nutritional and metabolic requirements; therefore, inappropriate feeding and deficiency of nutrients may lead to failure or malfunction of organs or tissues, and their inability to efficiently support functions such as keratinization of claw horn and immunological response to foot-related lameness caused by pathogens [19].

#### 3.2.3. Farming System

The farming system affects both the epizootiological traits and the major causes of foot-related lameness in dairy sheep. In arid and semi-arid areas, where extensive and semi-extensive farming systems prevail, injuries, interdigital pouch inflammation, and foreign bodies may be common causes of foot-related lameness [48]. Under these systems, horizontal transmission of footrot is likely to occur due to sharing of communal pastures by the flocks and is observed during the wet season, associated with the increased moisture in the ground and the extended housing under poor conditions. Depending on the type of terrain of the pasturelands, overgrowth of the hooves may be a significant cause of foot-related lameness [48]. In wet and soft terrains the hooves are not worn down adequately and, unless they are trimmed, they tend to overgrow, causing or predisposing to foot-related lameness. On the contrary, grazing in hard, stony terrains facilitates the natural wear down of the hooves and prevents them from overgrowing, keeping them in an appropriate shape and size. In low-input systems, inappropriate feeding may also play a vital role in the production of poor-quality claw horn, which predisposes to injuries and foot-related lameness.

In semi-intensive and intensive systems, footrot, WLD, and pedal joint abscess are the leading causes of foot-related lameness. Under these systems, sheep either remain housed or graze for short periods in nearby irrigated grasslands with soft and wet terrains. The major risk factors tend to be the overgrowth of hooves, the increased stocking density, and the inappropriate hygiene conditions inside the barn. Intensive feeding, with increased consumption of concentrates (to support a high milk yield potential), may lead to subacute ruminal acidosis and laminitis. Sheep, however, seem to be more resistant compared to cows [14,19,23,40].

#### 3.2.4. Farm Characteristics and Housing Conditions

Flock size and housing conditions can predispose to foot-related lameness in dairy sheep. In semi-intensive and intensive dairy sheep farms, the incidence of lameness has been found to be lower in bigger flocks [19]. This finding could be attributed to the increased know-how of big farms in utilizing the appropriate infrastructures and technologies. By incorporating modern husbandry and preventive veterinary practices, big farms exploit the benefits of scale economies to improve their flock health status. This does not seem to be the case in meat sheep in the UK, where no association between flock size and foot-related lameness occurrence has been observed [14]. In any case, it seems that differences between production systems do not allow a direct comparison of data from various sources. Thus, a universal conclusion cannot be postulated.

Among different housing conditions, increased moisture in the floor is the leading risk factor for the appearance of foot lesions and diseases causing foot-related lameness [19,23,40]. Elevated moisture in the floor results in reduction of claw horn hardness and increase in its size and weight [65]. Softer and swollen claws wear out rapidly due to friction, undermining their integrity and increasing the risk of foot-related lameness [66,67]. Consequently, inappropriate floor material and insufficient bedding are risk factors of foot-related lameness. Soft, smooth, and wet floors favor hoof overgrowth and water retention by the claw horn. Absence of floor stratification for appropriate drainage, insufficient exchange of bedding material, and poor ventilation lead to increased moisture on the ground, which in turn creates adverse conditions for hoof health [68]. In dairy sheep, except for the moisture on the floor, wet pasturelands, humid climate, and micro-climate in the barn may predispose to infectious diseases such as footrot and CODD [19,40].

Increased stocking density is a significant risk factor for the occurrence and the severity of foot-related lameness in dairy sheep, particularly in intensively reared and prolonged or permanently housed flocks. Housing space of <2 m^2^ per ewe was associated with a 2.3 times higher likelihood of developing lameness comparing to >2 m^2^ per ewe [19]. Overcrowding is usually followed by increased moisture and the accumulation of feces and urine in the bedding, compromising the hygiene status and favoring the proliferation and transmission of bacteria which cause footrot and CODD [37,69,70]. Moreover, the presence of urea and ammonia in the bedding causes molecular alterations in the keratin structure by breaking hydrogen and disulphide bonds, resulting in additional water absorption, swelling, and softening of claw horn with adverse effects in the hoof’s integrity [71].

#### 3.2.5. Inappropriate Foot Care

Foot care in sheep refers to claw trimming and footbathing. Foot over-trimming can cause injury and bleeding of the laminar corium and the development of granulomatous tissue at a later stage, predisposing to lameness. Moreover, inadequate disinfection of hoof trimming equipment promotes the transmission of pathogens such as *D. nodosus* within the flock and increases the incidence of foot-related lameness [12,15,23]. 

Footbathing is an efficient way to control foot infections. However, special attention needs to be paid to the correct application, the hygiene conditions, the concentration of the disinfectants used, and the time the animals stand in the footbath. When implementing footbathing, the feet need to be clean to allow penetration of the solution to the claw horn and possible sites of lesions; otherwise, the accumulation of organic matter inactivates the active substance of the solution and facilitates the proliferation and horizontal transmission of pathogens, further predisposing to infection [14,15,23,36]. For example, post-dipping lameness may result from the transmission of *Erysipelothrix rhusiopathiae* from infected to uninfected animals during footbathing.

#### 3.2.6. Inadequate Nutrition

##### Nutritional Deficiencies

In dairy sheep, nutritional demands for maintenance, growth, milk production, physical activity, and thermoregulation are increased compared to meat and wool sheep. Inappropriate feeding and nutritional deficiencies may deteriorate claw horn quality, making hooves more vulnerable to infections, lesions, and injuries. Therefore, the deficiency of specific nutrients predisposes to foot-related lameness.

The sulfur-containing amino acids cysteine and methionine are essential in supporting the structural and functional integrity of the hoof, as they participate in the formation of disulphide bonds during the keratinization of keratinocytes [72]. Normally, the rumen flora produces adequate amount of these amino acids when the sulfur content of the diet is appropriate. In some cases though, in high-yielding milking sheep, rumen production of these amino acids is inadequate to cover the high demand of lactation, even if an adequate quantity of sulfur is provided in the ration. In these cases, optimizing the rumen bypass protein fraction of the ration is necessary.

Linoleic and Arachidonic acids and Biotin (co-enzyme of Linoleate and Arachidonate metabolism) are also produced by the rumen flora and are critical for the integrity of the hoof, as they form a barrier against water loss from the claw horn, and they enhance the consistency and resilience of the hoof [73]. Vitamins A, C, and E protect the aforementioned fatty acids from oxidation [74]. Vitamin A is also essential for the differentiation of keratinocytes [30]. Among macro- and micro-elements, the deficiency of calcium, zinc, and copper negatively affect keratinization [75,76], while Selenium deficiency has been associated with susceptibility to footrot. Overall, deficiency of the aforementioned amino acids, fatty acids, vitamins, and macro- and micro-elements are associated with inferior claw horn quality and predisposition to foot-related lameness.

##### Imbalanced ratios

Systemic metabolic diseases, consumption of moldy feeds with mycotoxins, and fiber-deficient diets rich in starch and protein favor ruminal acidosis and predispose to laminitis in cattle [50,77]. Changes in the haemodynamics of corium, thrombosis, and ischaemia are the main causes that drive the pathogenesis of laminitis. They are usually triggered by endotoxins released during endotoxaemia, overproduction of lactic acid in the rumen, and histamine release after allergic reactions or directly absorbed by the intestine [46]. The occurrence of laminitis in meat and wool sheep is low. Despite the shortage of global and regional epidemiological data in dairy sheep, all evidence suggests that the disease is prevalent but likely under- or misdiagnosed.

#### 3.2.7. Insufficient Hygiene Measures 

Absence or insufficient hygiene measures predispose to foot-related lameness. The most critical omissions include: (i) Purchase of breeding stocks from flocks infected by footrot or CODD or with increased prevalence of foot-related lameness; (ii) lack of quarantine and regular examination of new-entry breeding stocks; (iii) inadequate disinfection of clothes, vehicles, trimming equipment, and pens used for lame animals; (iv) no separation of lame animals; and (v) maintenance of chronically infected, lame animals [37].

#### 3.2.8. Farmer’s Unawareness

The majority of farmers consider lameness as an important problem. Nevertheless, studies have shown [78,79] that some farmers are incapable of efficiently detecting foot-related lameness, whereas many of them do not clearly understand its etiology, the significance of predisposing factors, its epizootiological traits, and the value of the early implementation of appropriate preventive and control measures for the well-being of their animals and the sustainability of their operations [78,79,80]. In many cases, lack of training on the standard operating procedures regarding the early diagnosis and the implementation of footbaths and claw trimming seems to be the problem.

## 4. Diagnosis of Foot-Related Lameness

The first step towards effective control of foot-related lameness in sheep is early and proper diagnosis. In dairy sheep, early diagnosis of foot-related lameness can be achieved by observing or recording the animals walking in a single line before entering the milking parlor. In that case, diagnostic tools of lameness include direct gait assessment either through observation using appropriate locomotion score scales or state-of-the-art technologies, like sensors, 3D and thermographic cameras, and reaction-force measuring platforms. 

Locomotion scoring scales can be either numerical, using discrete values (five-degree and the seven-degree scales) [81,82] or analogue values in a 100 mm visual scale [83]. Other scales have been used to assess both locomotion score and the severity of lesions from footrot [84]. In all rating systems, the lowest score is assigned to normal posture and gait and the highest score in animals either unable to bear any weight in the affected leg during standing [81] or completely unable to stand and walk [82]. Locomotion scoring systems also serve as welfare assessment tools and indicators of lesion severity and pain [85]. From the assessment of between- and within- observer reliability in the same or different locomotion scoring systems, it can be concluded that locomotion scoring is more effective when performed by the same trained observer [40,82,83].

Sensors mounted within collars are currently available for measuring activity and behavior of sheep. These collars transmit data regarding the orientation, acceleration, and velocity of animals’ movements to interactive software, where machine learning-based algorithms are utilized to indicate locomotion and behavior patterns. Hence, automatic detection and recording of lameness and its severity can be achieved, and the most suitable recommendations for their treatment can be suggested on an evidential basis [86,87].

Computer-assisted vision techniques, exploiting 3D video systems, have been used in tracking posture and movement patterns in cows to recognize lame animals and evaluate their locomotion score in a precise way early on [88,89,90,91,92]. These systems offer an automated, non-invasive, welfare-friendly, and cost-effective way to monitor locomotion systematically. Nevertheless, despite their objectivity, more sophisticated machine learning techniques, including deep and recurrent learning ones, need to be integrated to increase their sensitivity and accuracy for lameness detection. Practical implementation of similar systems in sheep may be feasible, after appropriate modifications, given that the basic principles of their function are the same across species.

Infrared thermography (IRT) is another rapid and reliable point-of-care diagnostic tool for the initial allocation of inflammation sites in the feet, associated with the occurrence of lameness. In addition to that, IRT is increasingly used in cows for the detection of several diseases, such as bovine viral diarrhoea, foot and mouth disease, bluetongue, and mastitis [93]. Especially for foot inflammation, IRT is used to compare the temperature of the coronary band and foot skin within and between cows, providing a reliable diagnostic tool [94,95,96]. The diagnostic significance of IRT has been recently demonstrated in sheep [93,97]. The aforementioned research group [93] compared the application of IRT with standard measurements of temperature and oxidative stress biomarkers in sheep, and concluded that both of them could be applied in the early diagnosis of foot lesions and lameness in sheep. However, targeted adjustments, based on the ambient temperature and productivity stage, need to be implemented to improve the diagnostic capability of IRT in discriminating the occurrence and severity of foot-related lameness in sheep [97].

Reaction-force measuring platforms and pressure-sensitive walkaways have already been used in the study of animal gait, as they measure the forces (the first in an orthogonal way and the latter in a vertical way) from the legs to the ground and the weight distribution on four legs when the animal moves across them [98,99]. The pressure-sensitive walkway seems to be more convenient for gait assessment, as the vertical forces are more stable against the external parameters than the orthogonal ones, and the procedure is less time-consuming in a walkaway than in a force platform [98]. In sheep, the application of pressure-sensitive walkaway for lameness diagnosis seems to be possible according to researchers who succeeded in collecting reliable kinetic data according to spatial and temporal parameters [100].

## 5. Control of Foot-Related Lameness in Sheep

Strategies for the control of lameness in sheep may vary between and within the different production systems, and disagreements in the professional circle regarding the most efficient control of lameness do exist. However, there are some generally accepted principles which need to be considered when planning the farm’s foot-related lameness control plan [12]. Control of foot-related lameness in sheep is based upon the early and accurate diagnosis as described above, the evaluation and improvement of housing conditions, the appropriate modifications in feeding and nutrition, the targeted implementation of claw trimming and footbathing, the evidence-based planning of hygiene measures and preventive veterinary practices (i.e., antibiotic administration, vaccinations against footrot), the selective breeding to footrot resistance, and the training of the farmers and farm consultants.

### 5.1. Appropriate Housing Conditions and Feeding

Great attention needs to be given on the appropriate housing conditions for the elimination of foot-related lameness. Their significance is greater in intensively-reared dairy sheep, which remain permanently housed throughout their life [19]. Appropriate ventilation, stocking density, floor, bedding material, and cleaning, followed by the disinfection of the premises are all crucial parameters for the prevention of lesions and diseases causing foot-related lameness [19,22,40]. The appropriateness of the aforementioned parameters is determined by the farmer and the consultant on an evidential basis, according to the peculiarities of the farmer, the farm, and the animals. In any case, the objective is to achieve hygienic housing conditions, with decreased moisture, urea, and manure accumulation in the hooves [37,68,69].

Rational feeding ensures that the animals are capable of meeting their nutritional demands in every stage of their production life and under any circumstances. To ensure the integrity and functionality of the hooves, the efficient immunological response and rumen’s health, well-balanced rations are indispensable to cover requirements of basic components; namely, necessary amino acids, fatty acids, vitamins, and macro- and micro-elements [19,22,46]. Sufficiency of the nutrients mentioned above is associated with good-quality claw horn and thereby, protection against foot-related lameness.

### 5.2. Targeted Implementation of Claw Trimming and Footbathing

#### 5.2.1. Footbaths

The application of footbaths is one of the popular control measures for footrot, OID, and CODD. However, appreciation by sheep farmers is variable [18,101,102]. In general, footbaths are more effective in curing mild infections rather than severe. The most commonly used disinfectants are solutions of zinc sulfate (10 to 20%), copper sulfate (5%) and formalin (3 to 5%). The concentration of the solution depends on the specific cause of foot-related lameness and the severity of the infection at flock level. Formalin footbaths are cheap and effective within a single application to animals. Currently, the trend is to abolish the use of formalin due to toxicity and its adverse effects on animal and human health. Zinc sulphate footbaths are widely advocated as an effective and painless treatment and one that remains efficient in the presence of organic matter. Τheir disadvantages include the costly implementation, the necessity to stand sheep in the solution for 2 to 30 min rather than walk through it, and the decreased solubility of zinc sulphate (surfactants need to be added). A copper sulphate footbath is effective and cheap; its solubility is satisfying and remains active at the presence of organic matter. However, it hardens the claw horn, and if not appropriately implemented, it may lead to copper toxicity when consumed by sheep. Footbaths with antibiotic solutions may also be efficient; however, they are not recommended as they can lead to increased antibiotic resistance, and require proper disposal, which is unlikely in most of the cases.

The standard footbath procedure consists of three consecutive stages. Initially, a first passage of animals through a clean water bath is necessary to remove mud and manure from the hooves that will allow the subsequent bath solution to penetrate the claw corn and the sites of infection. The second stage refers to the passage of animals through the footbath solution containing the disinfectant (filled up to 5–6 cm in the bath). Duration of this stage depends on the disinfectant used and the severity of the infection. The third stage is the post-bath drying process where the animals must remain on a hard and clean floor until the hooves are dry. Footbaths should be constructed in a hallway, with gates available at both ends, to facilitate keeping animals in the footbath solution for the required time. The total wall height, the length and width of the footbath need to be at last 50, 240, and 120 cm, respectively; the dimensions may be adjusted according to the number of animals and the frequency of footbaths. Footbathing facilities must be impervious to liquids, maintained in good condition, and disinfected regularly; whereas, the solution of the footbath has to be clean, frequently replaced, and appropriately disposed of according to relevant regulations for the protection of the environment [102]. Application of footbaths during dry and hot season gives good therapeutic results.

#### 5.2.2. Claw Trimming

Claw trimming in meat and wool sheep is recommended on a limited case-by-case basis, depending on the occurrence of overgrown hooves and foot-related lameness [12]. In intensively-reared dairy sheep, frequent claw trimming (once to twice per year), initiating before the first mating, may be necessary for the removal of overgrown claw horn and foreign bodies trapped inside. Routine feet inspection is also required for the early detection and treatment of defects and lesions which might lead to lameness. In that case, functional claw trimming restores the ideal shape of the hoof and improves the gait of the animal. In pastoral farming systems, claw trimming may not be necessary due to the normal wear down of the claws during grazing. In cases of foot-related lameness due to footrot, OID, and CODD, claw trimming can be used in some animals for diagnostic purposes but is not recommended as a standard therapeutic intervention, given that claw trimming facilitates further spreading of the pathogens and aggravates lesions in the hooves. In this regard, disinfection of claw trimming equipment is essential to avoid the transmission of infectious agents. Disinfection of the equipment during and after claw trimming and clearing up and appropriate disposal of trimmings are important for the prevention of the transmission of *D. nodosus* [12]. Claw trimming requires some skill and must always be implemented by trained personnel that adhere to standard operating procedures. In some cases claw trimming can be painful due to existent foot lesions, demanding the administration of analgesia by a veterinarian. Excessive claw trimming should be avoided, because it can cause injuries of the corium lamina and spreading of common pathogens.

### 5.3. Vaccination 

Administration of vaccines against footrot can be both a therapeutic and preventive strategy [103,104]. However, it is not always totally effective and usually elicits an immunity of relatively short duration [12]. For this reason, it needs to be repeated every six months or sooner. Currently, monovalent, bivalent, and multivalent vaccines are commercially available, containing either whole cell antigens or fimbrial antigens of *D. nodosus*. Among them, the most commonly used are the multivalent vaccines, which contain all of the ten recognized serogroups of *D. nodosus* (serogroups A to I and M) [103]. They offer adequate immunization for about 10 weeks, whereas, the monovalent and bivalent vaccines offer longer protection but only from infections of the corresponding serotypes. Monovalent vaccines have been used in eradication programs, where strains from a single serogroup have been found to be the causative agent of footrot (Australia, Nepal, Bhutan). The greatest challenge in immunization against footrot is to develop vaccines which will provide longer immunity against a higher range of strains, via the identification and use of a common antigen for all serogroups [104].

### 5.4. Antibiotics

Administration of antibiotics can be performed either on an ad-hoc individual basis or a whole-flock basis for treatment and prevention of transmission of pathogens causing foot-related lameness, in accordance to national regulations [105]. Topical administration of Oxytetracycline spray can be effective in treating early stages of footrot, as well as for the treatment of OID [12,101]. Injectable forms of long-acting Oxytetracycline are indicated in more severe cases of footrot as well as CODD [101,104,106,107]. Other commonly used antibiotics in meat and wool sheep include Penicillin/Streptomycin [12,108], Lincomycin/Spectinomycin [109], Erythromycin [110], Tylosin [111], Tilmicosin [12], and Gamithromycin [107]. In dairy sheep, the list of the antibiotics used is limited due to legal limitations and extended withdrawal period of milk, although in some cases, off-label use of Ceftiofur is practiced. In any case, after the administration of injectable antibiotics, the animals should be transferred to a clean, appropriately disinfected, and dry environment [20,39], with summer being the ideal season for the control of footrot, OID, and CODD. NSAIDs like Flunixin Meglumine have not been found to have an explicit effect on recovery from footrot [106]; however, their efficacy needs to be further investigated.

### 5.5. Hygiene Measures 

To prevent transmission of pathogens causing foot-related lameness and provide better veterinary services, lame animals need to be separated from the rest of the flock until healthy. In addition, bought-in sheep need to be quarantined for at least two weeks to prevent the introduction of footrot and CODD in the flock. Culling of chronic lame animals and replacement of breeding stocks from footrot-free sheep farms have to be integral parts of prevention, control, and eradication programs [58]. In the case of grazing flocks, pasturelands which have been free of sheep for at least two weeks are not likely to promote *D. nodosus* infection, as this bacterium is incapable of surviving beyond the claw horn for more than seven days.

### 5.6. Breeding for Resistance to Footrot

Breeding for resistance to footrot is a potentially feasible option, as a commercial genetic marker test is available. It has been developed in New Zealand and it is currently used for the selection of resistant animals without the involvement of clinical disease and phenotypic assessment [112,113]. The results from the application of the genetic test are satisfactory and its economic benefits have been estimated to be enormous for the sheep industry in New Zealand. This test is based on the detection of DQA2-specific alleles to classify sheep according to their resistance or susceptibility to footrot [112,113]. However, it has been developed in specific breeds and it remains rather unexploited worldwide, as its applicability in other breeds (including dairy breeds), although possible, has not been sufficiently evaluated yet [19,58]. A more thorough investigation of the existing or newly discovered alleles and genotypes, as well as an assessment of their association with footrot susceptibility, need to be applied in a larger scale including more breeds and production systems [19].

Genetic selection based on the phenotype and the severity of clinical signs of footrot can also be used for the long-term control of the disease [113], as the inheritance factor of footrot varies from 0.15 to 0.25 and hence, it is likely to be useful in animal selection [55]. Similarly, genetic selection to footrot can be achieved through the continuous culling and replacement of severely infected sheep in farms with high prevalence rates of the disease [58]. Genetic selection may also be applied for WLD in the future, when the involving alleles and the possible genetic correlation with footrot susceptibility will be clarified [64].

## 6. Conclusions

The aetiologic infectious agents involved in pathogenesis of foot-related lameness, the factors involved in their transmission, as well as the establishment and clinical manifestation of relevant diseases in sheep are well known. Foot-related lameness is primarily an animal husbandry-oriented disease and therapeutic strategies should only be viewed as a mean to minimize symptoms and limit animal discomfort. Preventive hygiene measures to limit introduction of pathogens in the flock (quarantine of incoming animals) and block transmission (vaccines, foot baths) should be at the for front of the standard health management system. Frequent claw trimming, proper nutrition and appropriate floor conditions aid in maintaining tissue integrity and prevent pathogen invasion in the affected areas. Dairy sheep in semi-intensive and intensive rearing systems seem to be more vulnerable to foot-related lameness. Proper education of farmers and training of associated personnel by professional health care providers in these operations is critical for the sustainability and profitability of the farms and the well-being of animals.
